# Structural evidence for the binding of monocarboxylates and dicarboxylates at pharmacologically relevant extracellular sites of a pentameric ligand-gated ion channel

**DOI:** 10.1107/S205979832000772X

**Published:** 2020-06-30

**Authors:** Zaineb Fourati, Ludovic Sauguet, Marc Delarue

**Affiliations:** a Unité Dynamique Structurale des Macromolécules, Institut Pasteur, 25 Rue du Docteur Roux, F-75015 Paris, France; b Centre National de la Recherche Scientifique, CNRS UMR3528, Biologie Structurale des Processus Cellulaires et Maladies Infectieuses, 25 Rue du Docteur Roux, F-75015 Paris, France

**Keywords:** pentameric ligand-gated ion channel, GLIC, carboxylates, orthosteric site, vestibular site

## Abstract

GLIC is a bacterial homologue of the pentameric ligand-gated ion channels (pLGICs) that mediate the fast chemical neurotransmission of nerve signalling in eukaryotes. Because the activation and allosteric modulation features are conserved among prokaryotic and eukaryotic pLGICs, GLIC is commonly used as a model to study the allosteric transition and structural pharmacology of pLGICs. It has previously been shown that GLIC is inhibited by some carboxylic acid derivatives. Here, experimental evidence for carboxylate binding to GLIC is provided by solving its X-ray structures with a series of monocarboxylate and dicarboxylate derivatives, and two carboxylate-binding sites are described: (i) the ‘intersubunit’ site that partially overlaps the canonical pLGIC orthosteric site and (ii) the ‘intrasubunit’ vestibular site, which is only occupied by a subset of the described derivatives. While the intersubunit site is widely conserved in all pLGICs, the intrasubunit site is only conserved in cationic eukaryotic pLGICs. This study sheds light on the importance of these two extracellular modulation sites as potential drug targets in pLGICs.

## Abstract 2

Co-crystal structures of GLIC, a bacterial ligand-gated ion channel, in complex with monocarboxylate and dicarboxylate derivatives are reported. It is shown that binding occurs at two pharmacological sites in the extracellular domain, which is in agreement with the reported effect of some carboxylates as allosteric modulators of GLIC.

## 1. Introduction   

Pentameric ligand-gated ion channels (pLGICs) are key receptors in the nervous system that mediate nerve signal propagation throughout the synapse. A variety of pharmacological molecules modulate pLGIC activity and thus modify their response towards their respective agonists. The unexpected occurrence of pLGIC orthologues in prokaryotes was discovered only a decade ago (Tasneem *et al.*, 2005 ▸). Shortly afterwards, functional studies established that their activation and modulation mechanisms are similar to those in eukaryotes (Zimmermann & Dutzler, 2011 ▸; Weng *et al.*, 2010 ▸). Therefore, thanks to their better disposition for structural studies, prokaryotic pLGICs were used as structural models for the characterization of the transition and dynamics of pLGICs, with the best characterized being that from *Gloeobacter violaceus*, which is named GLIC (Bocquet *et al.*, 2007 ▸). Indeed, the structure of GLIC has been solved in several conformational states, including an apparently open state (Bocquet *et al.*, 2009 ▸; Sauguet, Poitevin *et al.*, 2013 ▸; Fourati *et al.*, 2015 ▸), a resting-like state (Sauguet *et al.*, 2014 ▸) and an intermediate locally closed state (Prevost *et al.*, 2012 ▸), and in complexes with a range of pharmacological molecules, including general anaesthetics (Fourati *et al.*, 2017 ▸, 2018 ▸; Laurent *et al.*, 2016 ▸; Sauguet *et al.*, 2016 ▸; Sauguet, Howard *et al.*, 2013 ▸; Kinde *et al.*, 2016 ▸; Hilf *et al.*, 2010 ▸; Zimmermann *et al.*, 2012 ▸). GLIC thus arose as a suitable tool to characterize the structural transition occurring during activation of pLGIC and its modulation at the molecular level.

Unlike the other pLGICs, GLIC displays a particular activation mechanism in which protons, rather than a sizable agonist, trigger ion-channel opening. We have recently characterized another bacterial pLGIC displaying a comparable response to pH, albeit with an opposite sensitivity, where receptor activation is triggered by alkaline pH (Hu *et al.*, 2018 ▸). This latter receptor from a tubeworm symbiont bacterium, named sTeLIC, is strongly potentiated by a cinnamic acid derivative that binds at a specific vestibular site of the extracellular domain located behind the common pLGIC orthosteric site (Hu *et al.*, 2018 ▸). Interestingly, GLIC is also modulated by cinnamic acid derivatives as well as other carboxylic acid derivatives such as caffeic and crotonic acids, which display consistent inhibition of GLIC currents (Prevost *et al.*, 2013 ▸; Alqazzaz *et al.*, 2016 ▸). While these compounds have been suggested to bind at the pLGIC orthosteric site in GLIC by docking studies, no experimental data are yet available to support this hypothesis. On the other hand, we have also identified two acetate-binding sites in the X-ray structure of GLIC in the apparently open state, where acetate was used as a buffering agent in the crystallization solution (Sauguet, Poitevin *et al.*, 2013 ▸; Fourati *et al.*, 2015 ▸): (i) a vestibular site similar to the bromocinnamic acid site in the sTeLIC structure and (ii) a site partially overlapping with the canonical pLGIC orthosteric site. These sites were called the ‘intrasubunit’ and ‘intersubunit’ acetate sites, respectively, according to their localization within or between the receptor subunits. Our rationale for this study was to provide experimental evidence for the binding of carboxylate derivatives other than acetate to GLIC, especially those for which the modulation potency has previously been described, namely caffeic acid and crotonic acid (Alqazzaz *et al.*, 2016 ▸; Prevost *et al.*, 2013 ▸). While all of our attempts to crystallize GLIC with caffeic acid yielded only poorly diffracting crystals, we managed to solve the structure of GLIC in complex with crotonic acid, as well as with a series of other carboxylate derivatives (Table 1 ▸). We show that all of the carboxylate derivatives that we were able to co-crystallize with GLIC bind in the intersubunit site that partially overlaps the common pLGIC orthosteric site, and that only acetate, propionate and succinate bind in both the intersubunit and intrasubunit sites.

## 2. Methods   

### 2.1. Protein expression and purification   

The expression and purification process was performed as described previously (Bocquet *et al.*, 2009 ▸; Sauguet, Howard *et al.*, 2013 ▸; Sauguet, Poitevin *et al.*, 2013 ▸). Briefly, GLIC fused to maltose-binding protein was expressed in *Escherichia coli* C43 cells and solubilized from membranes using *n*-dodecyl-β-d-maltoside detergent. The protein was then subjected to affinity purification on amylose resin and, after maltose-binding protein cleavage, was further purified by size-exclusion chromatography, with elution in 300 m*M* NaCl, 20 m*M* Tris–HCl, 2 g l^−1^ *n*-dodecyl-β-d-maltoside pH 7.6, and concentration using a 100 kDa cutoff filter.

### 2.2. Crystal preparation   

All crystals were obtained by vapour diffusion in hanging drops at 18°C. The concentrated protein solution (10 g l^−1^, corresponding to a GLIC pentamer concentration of 55 µ*M*), was mixed in a 1:1 ratio with a reservoir solution consisting of 16% (2.2 *M*) glycerol, 12–14.5% (34–41 m*M*) polyethylene glycol 4000, 2% (0.28 *M*) dimethyl sulfoxide, 400 m*M* sodium thiocyanate with the buffering carboxylic acid adjusted to pH 4 using NaOH–HCl. The concentration of the monocarboxylic or dicarboxylic acid compound used as a buffer in the reservoir solution was 100 m*M*, except for fumarate (30 m*M*) owing to a solubility issue. The quality of the crystals was improved by the microseeding technique: a suspension of crushed crystals grown in the same crystallization condition but in the presence of acetic acid/acetate was added upon setting up the crystallization experiment. Crystals appeared overnight with a parallelepiped-like shape and grew for one week before reaching their final dimensions. All of the crystals were directly flash-cooled in liquid nitrogen prior to data collection.

### 2.3. Data collection   

X-ray data sets were collected on the PROXIMA 1 and PROXIMA 2 beamlines at the SOLEIL synchrotron, Gif-sur-Yvette, France and on the ID23-1 and ID29 beamlines at the European Synchrotron Radiation Facility (ESRF), Grenoble, France. Reflections were integrated using *XDS* (Kabsch, 2010 ▸) and further processed using programs from the *CCP*4 suite (Winn *et al.*, 2011 ▸). As expected, crystals of GLIC grown at pH 4 were isomorphous to the previously described crystal lattice of the open-pore receptor and belonged to space group *C*121 (unit-cell parameters *a* = 113, *b* = 127, *c* = 185.8 Å, α = γ = 90, β = 101°) with one pentamer in the asymmetric unit, with the exception of the GLIC–succinate structure, in which two pentamers, oriented back to back, were found in the asymmetric unit (unit-cell parameters *a* = 113, *b* = 127, *c* = 320 Å, α = γ = 90, β = 101°).

### 2.4. Phasing and refinement   

The phases were directly calculated by performing rigid-body refinement with *REFMAC*5 (Murshudov *et al.*, 2011 ▸) using PDB entry 3eam (Bocquet *et al.*, 2009 ▸) as a starting model. The structure was then subjected to restrained refinement with *REFMAC*5 using noncrystallographic symmetry restraints. The resulting model was improved by manual building in *Coot* (Emsley *et al.*, 2010 ▸) and was subsequently refined using *BUSTER* (Blanc *et al.*, 2004 ▸). The final structure was validated using the *MolProbity* web server (Chen *et al.*, 2010 ▸). The data-processing and refinement statistics are presented in Table 2 ▸.

## 3. Results   

### 3.1. Crystal structure of GLIC in complex with propionate   

**Figure 1:**
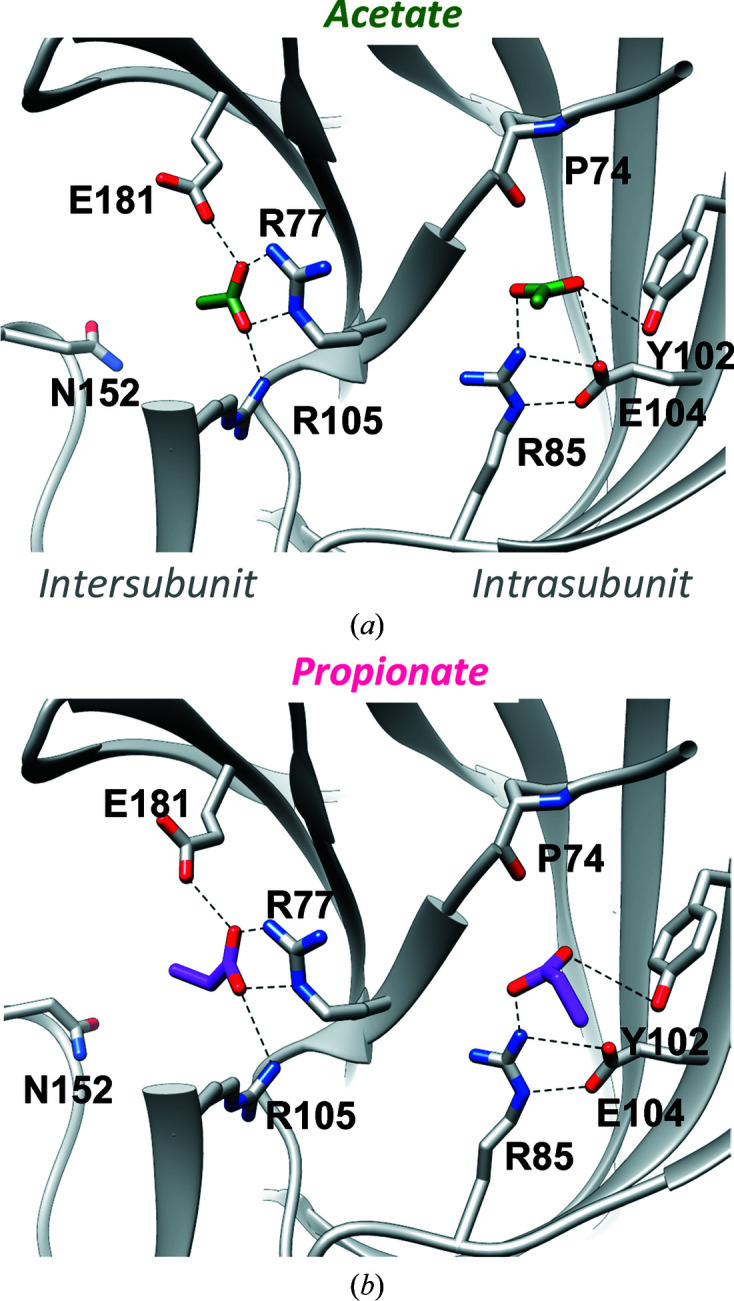
GLIC bound to the monocarboxylic acids acetate and propionate. (*a*) The acetate-binding pockets of the GLIC crystal structure (PDB entry 4hfi; Sauguet, Poitevin *et al.*, 2013 ▸), shown in a detailed view with the intersubunit pocket on the left and the intrasubunit pocket on the right (acetate molecules in green-coloured stick representation). Five such pairs of intersubunit and intrasubunit sites are present in the GLIC homopentamer. (*b*) Similar view of a propionate-bound GLIC crystal structure, showing that propionate molecules (pink) are present in the same intersubunit and intrasubunit sites in GLIC. Note the similar protein conformation in (*a*) and (*b*).

We previously identified ten acetate-binding sites (two per monomer) within the high-resolution GLIC X-ray structure obtained from crystals grown at pH 4 in acetate buffer (Bocquet *et al.*, 2007 ▸). These acetate molecules occupied five homologous intersubunit sites and five homologous intra­subunit sites (Fig. 1 ▸ *a*). Here, we solved a 3.2 Å resolution structure of GLIC in complex with propionate obtained from crystals grown in a solution buffered at pH 4.0 using 100 m*M* propionate instead of acetate. Similarly to acetate, propionate molecules were found both in the intrasubunit site, where propionate interacts with the side chains of Tyr102 and Arg85, and in the intersubunit site at the bottom of the agonist site, where propionate interacts with Glu181 and Arg77 of one subunit and with Arg105 of the complementary subunit (Fig. 1 ▸ *b*). The overall structure was similar to the acetate-bound structure (root-mean-square deviation of below 0.3 Å calculated over 310 residues), and no significant remodelling of the intrasubunit and intersubunit pockets was observed upon propionate binding.

### 3.2. Crystal structure of GLIC in complex with succinate   

**Figure 2:**
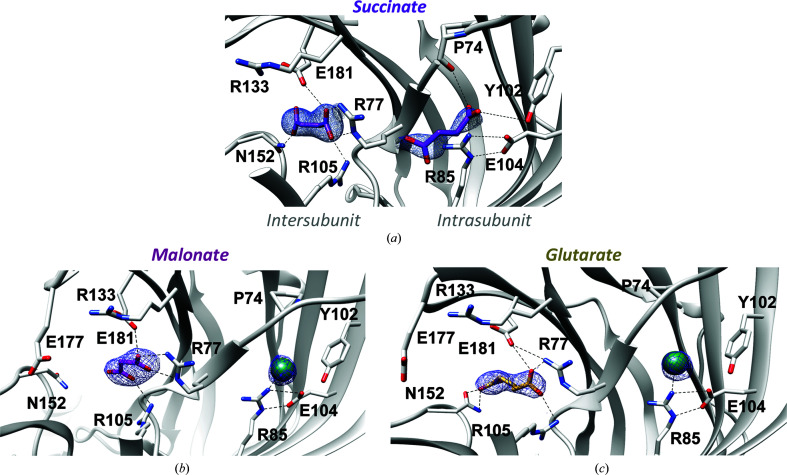
GLIC crystal structures with dicarboxylates: succinate (*a*), malonate (*b*) and glutarate (*c*). (*a*) Detailed view of the succinate-bound GLIC structure, showing both intersubunit (left) and intrasubunit sites occupied by succinate molecules (purple sticks). (*b*, *c*) Similar views of the carboxylate-binding pockets in crystal structures of GLIC in complex with malonate (*b*) and glutarate (*c*). Only the intersubunit site is occupied by a molecule of malonate (pink) or glutarate (dark yellow). The intrasubunit pocket is occupied by a chloride ion (green sphere) in both cases. The 2*F*_o_ − *F*_c_ density contoured at 2 Å around the ligands is coloured blue and contoured at 1σ.

So far, only monocarboxylic acids have been reported to bind GLIC [acetic (Fourati *et al.*, 2015 ▸), caffeic, cinnamic (Prevost *et al.*, 2013 ▸) and crotonic acids (Alqazzaz *et al.*, 2016 ▸)]. We thus aimed to check whether dicarboxylic acids are also able to bind GLIC similarly to monocarboxylates. A 2.5 Å resolution structure was obtained from crystals grown in a solution buffered at pH 4.0 using 0.1 *M* succinate (diC4). In this structure, two identical GLIC pentamers were found in the asymmetric unit, with a *c* unit-cell parameter that was twice as large as in the other structures described in this study (Supplementary Fig. S1*a*). Strong *F*_o_ − *F*_c_ densities, reminiscent of succinate binding, were observed in the intersubunit and intrasubunit sites, and succinate was modelled in both sites previously described to bind acetate or propionate (Fig. 2 ▸ *a*). The succinate molecule bound in the intersubunit site is modelled with one of its carboxylate moieties superimposed on the position of acetate below Loop C. This carboxylate moiety interacts with the side chains of residues from two adjacent subunits: Arg77 (Loop A) and Glu181 (Loop C) from one subunit and Arg105 from the neighbouring subunit. The other carboxylic moiety of succinate interacts with the side-chain amino group of Asn152 from the complementary subunit (Fig. 2 ▸ *a*).

The succinate molecule in the intrasubunit site displayed a higher flexibility than the intersubunit succinate, as seen in the different subunits. Indeed, the first succinate carboxylic moiety binds in the same way (in all subunits) as acetate: through a hydrogen bond to the hydroxyl group of the well conserved Tyr102 and a salt bridge to the guanidinium group of Arg85, which itself makes a salt bridge with Glu104. However, the second carboxylic moiety of the intrasubunit succinate molecule only interacts with the main-chain carbonyl group of Pro74, leading to different binding poses of succinate in the intrasubunit site in the different subunits (Supplementary Fig. S1*b*).

This analysis thus reveals new residues that are involved in carboxylate binding, namely the side chain of Asn152 in the intersubunit pocket and the main chain of Pro74 in the intrasubunit pocket (Supplementary Fig. S1*c*).

### 3.3. Crystal structures of GLIC in complex with malonate and glutarate   

In attempt to check whether other dicarboxylic acids with different lengths are also able to bind GLIC, we solved crystal structures of GLIC in complex with malonate (diC3) and glutarate (diC5) at 2.9 and 2.65 Å resolution, respectively. Surprisingly, densities corresponding to malonate and glutarate were only found in the intersubunit sites, whereas a globular density, modelled as a chloride ion, was found in the intrasubunit sites of both structures (Figs. 2 ▸ *b* and 2 ▸ *c*). The first carboxylic groups of malonate and glutarate occupied the position of acetate, with a similar interaction pattern with Arg77, Glu181 and Arg105. The second carboxylic group of glutarate forms a hydrogen bond to Asn152, as for succinate, whereas that of malonate interacts with the side chain of Arg105 (Figs. 2 ▸ *b* and 2 ▸ *c*)

### 3.4. Crystal structures of GLIC in complex with crotonate and fumarate   

**Figure 3:**
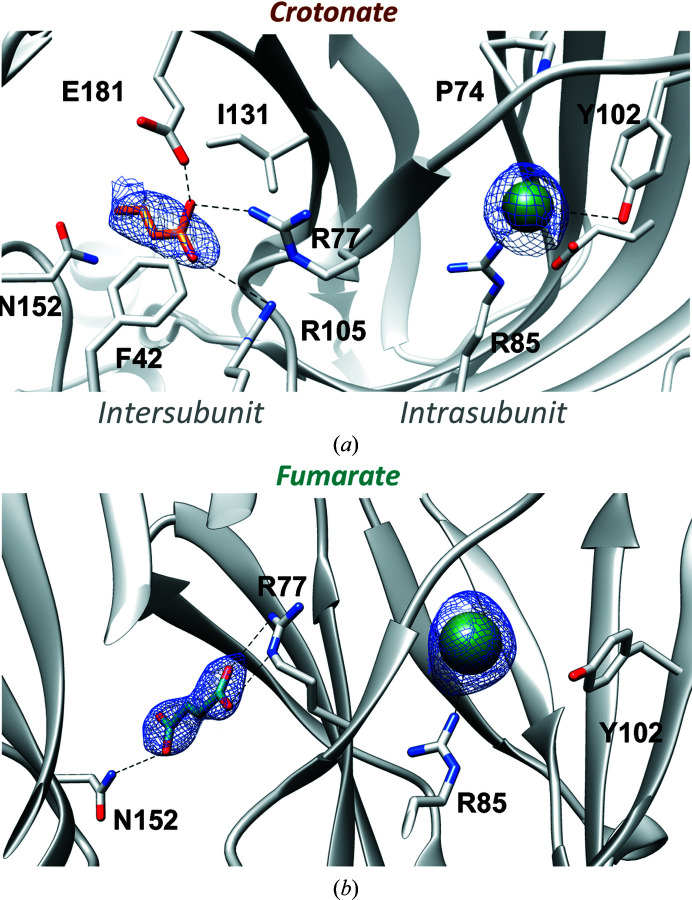
GLIC crystal structures with the unsaturated C4 carboxylates crotonate and fumarate. Crystal structures of GLIC in complex with (*a*) crotonate (orange stick representation) and (*b*) fumarate (dark cyan). Both C4 compounds with a double bond in the *trans* configuration are present only in the intersubunit site, while the intrasubunit pocket is occupied by a chloride ion (green sphere) in both structures. The 2*F*_o_ − *F*_c_ density contoured at 2 Å around the ligands is coloured blue and contoured at 1σ.

We then tested for GLIC binding by unsaturated carboxylic acids. Using the *trans* isomer crotonate (monoC4) at 0.1 *M* as a buffer produced co-crystals of GLIC that provided a 2.9 Å resolution structure in which a crotonate molecule occupied the intersubunit pocket, whereas the spherical density in the intrasubunit pocket was modelled as a chloride ion (Fig. 3 ▸ *a*). The crotonate molecule was oriented along the axis between Arg77 and Asn152, as was observed for the succinate molecule present in the intersubunit pocket. This orientation partially overlaps with that previously described by molecular docking (Alqazzaz *et al.*, 2016 ▸); in particular, Ile131 from the principal subunit and Phe42 from the complementary sububnit are in the vicinity of the hydrophobic part of crotonate (side chains at 4.6 and 3.4 Å, respectively, from the crotonate C2 atom) and could thus partially contribute to its coordination. However, Arg133 is not involved in direct crotonate coordination, although it can mediate Glu181 carboxyl stabilization through hydrogen bonding (Alqazzaz *et al.*, 2016 ▸).

**Figure 4:**
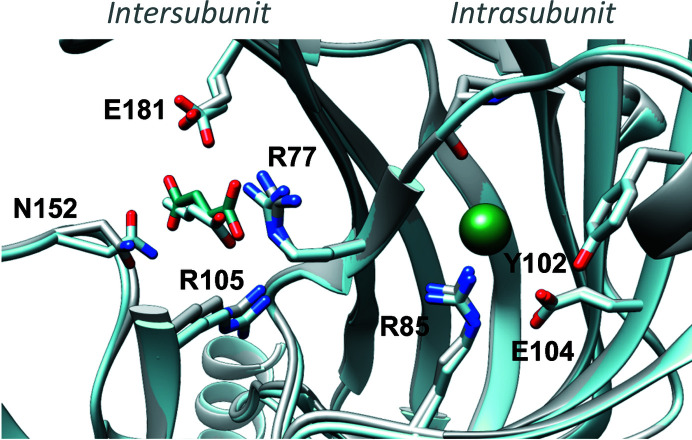
Comparison of GLIC structures with crotonate and fumarate. The GLIC–crotonate structure (cyan) is superimposed on the GLIC–fumarate structure (grey), with fumarate shown as dark cyan sticks, crotonate as cyan sticks and chlorine as a green sphere.

The *trans* isomer fumarate (diC4) was then used as a buffering agent at 0.03 *M*, producing a 2.9 Å resolution structure. A very similar orientation was adopted by fumarate in the intersubunit pocket, whereas a chloride ion again occupied the intrasubunit pocket (Fig. 3 ▸ *b*). The first carboxylic acid moiety occupied the same position as acetate, near Arg77 and Arg105, and the C4 atom could be superimposed on the C4 atom of crotonate. The distances between the C4 atom and Glu181 or Asn152 were unchanged in the fumarate and crotonate structures, although Asn152 is involved in the coordination of fumarate but not crotonate through hydrogen bonding (Fig. 4 ▸).

The statistics of data processing and refinement are summarized in Table 2 ▸.

## 4. Discussion   

This work provides structural evidence of carboxylic acid binding to GLIC, as previously characterized by several functional studies. Although the carboxylates described to modulate GLIC (caffeic and cinnamic acid derivatives and crotonic acid) are actually allosteric inhibitors, all of the structures presented in this work show that they (also) bind the apparently open conformation of this ion channel. In particular, crotonic acid was described to be an inhibitor of GLIC, with an IC_50_ of 620 µ*M*. It is however noteworthy that GLIC inhibition by crotonic acid was measured at pH 5.5. In the crystallization condition (pH 4), the proton concentration thus might be too high to allow the equilibrium to shift towards channel closure, even at 100 m*M* crotonate. At the molecular level, the intersubunit site that partially overlaps with the common pLGIC orthosteric site appears to be the principal binding site for carboxylates. Indeed, all of the monocarboxylic and dicarboxylic acids were found to bind in this pocket with a similar binding mode for the first carboxylic group involving Arg77 and Glu181 from one subunit and Arg105 from the neighbouring sububnit. For dicarboxylates, except for malonate, Asn152 from the complementary subunit contributes to the coordination of the second carboxylic group. For malonate, the molecule is too short to reach the Asn152 side chain and the second carboxylate is thus coordinated by Arg105. In the crotonate structure, the hydrophobic tail is partially coordinated by weak hydrophobic interactions involving Ile131 and Phe41. These latter residues have been suggested to be involved in crotonate-mediated inhibition of GLIC currents (Alqazzaz *et al.*, 2016 ▸). Interestingly, this intersubunit site has also been described as a benzodiazepine-binding site in ELIC and a negative modulation potency was also attributed to it (Spurny *et al.*, 2012 ▸). This further suggests that this pocket is likely to be a relevant pharmacological site within GLIC as well as other pLGICs. However, we could not rule out the possibility that other sites can accommodate carboxylates within the resting conformation of the receptor and be responsible for inhibition.

**Figure 5:**
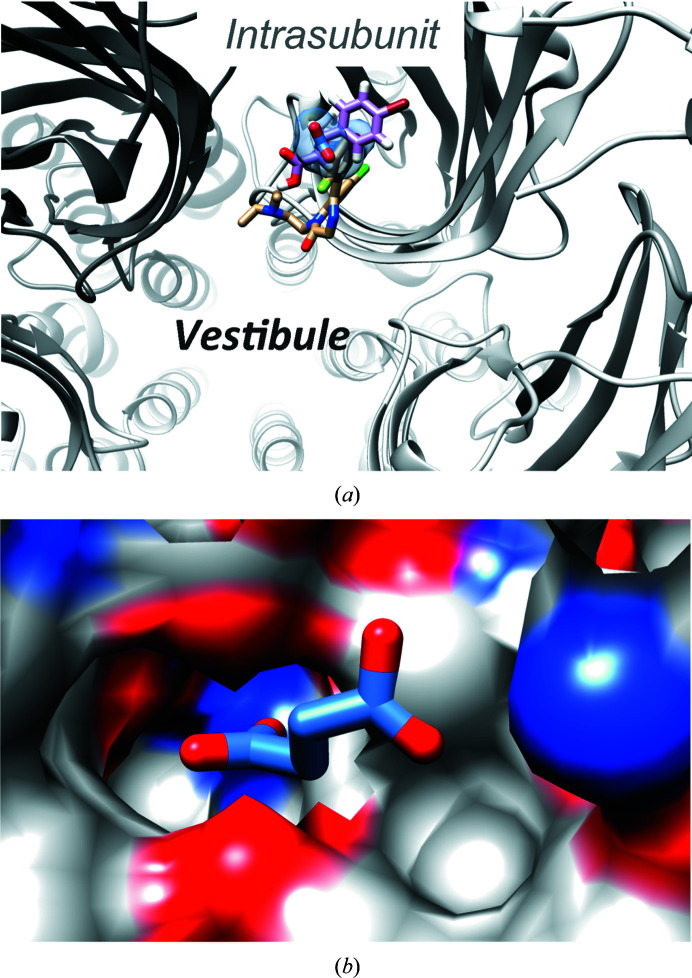
The intrasubunit site in other pLGICs. (*a*) Superimposition of the binding pose of succinate (blue sticks, blue surface) in the intrasubunit site of GLIC with those of flurazepam in ELIC (PDB entry 2yoe; Spurny *et al.*, 2012 ▸) coloured tan and bromocinnamic acid in sTeLIC (PDB entry 6fli; Hu *et al.*, 2018 ▸) coloured pink. (*b*) The succinate binding pose in GLIC superimposed on the intrasubunit pocket of the α4β2 acetylcholine receptor (PDB code 5kxi; Morales-Perez *et al.*, 2016 ▸). The surface representation was generated with *UCSF Chimera*.

Besides this sub-orthosteric intersubunit site, we show here that the intrasubunit site can accommodate some of the carboxylates described in this study, namely propionate and succinate (Supplementary Fig. 2*b*). Why these carboxylate derivatives bind in this intrasubunit pocket while others do not is not completely obvious, at least based on the co-structures. Indeed, simple docking experiments with fumarate show that it could also bind to the intrasubunit site. Crotonic acid, which is a monocarboxylic derivative of fumarate, might be unable to stably bind the intrasubunit site because of the absence of the second carboxylic group and be easily replaced by chloride. It is also noteworthy that succinate has higher *B* factors (mean intersubunit *B*_succinate_ of 85.1 Å^2^ versus a mean intrasubunit *B*_succinate_ of 106.5 Å^2^) in the intrasubunit binding site than in the intersubunit binding site, as well as a higher variability of binding poses within the intrasubunit site (Supplementary Fig. S1*b*). Unlike the intersubunit succinate, where both carboxylic groups are tightly coordinated through hydrogen bonds to Arg85, Tyr102 and Glu104, the second carboxylic group of succinate in the intrasubunit site points almost freely towards the solvent and is only loosely coordinated through a hydrogen bond to the main chain of Pro74 in most of the modelled succinate poses. This flexibility might explain why fumarate, which is a *cis*-unsaturated (and thus more rigid) derivative of succinate, was not seen experimentally in the intrasubunit site. From a functional point of view, this intrasubunit site, which is also called the vestibular site, has already been described as a potent modulation site in other pLGICs. Indeed, along with the intersubunit site, the vestibular intrasubunit site has also been described as a benzodiazepine-binding site (Spurny *et al.*, 2012 ▸). Interestingly, flurazepam potentiates ELIC at moderate concentrations but becomes an inhibitor at higher concentrations (over 200 µ*M*). The authors correlate this bimodal modulation with intrasubunit versus intersubunit site binding, where flurazepam would promote ELIC potentiation when bound to the ‘high-affinity’ intra­subunit site and inhibition, at higher concentrations, when bound to the ‘low-affinity’ intersubunit site. Besides ELIC, sTeLIC, a receptor that we have recently characterized, is also positively modulated by cinnamic acid derivatives that bind to the intrasubunit vestibular site with a similar potency as flurazepam in ELIC (EC_50_ of 21 µ*M* versus 12.5 µ*M* for flurazepam; Hu *et al.*, 2018 ▸). Both flurazepam and bromocinnamic acid in the vestibular sites of ELIC and sTeLIC, respectively, partially overlap with succinate bound to the GLIC intra­subunit site. However, bromocinnamic acid is more buried, with its bromophenyl ring pointing to the bottom of the cavity, while flurazepam extends further to the vestibule (Fig. 5 ▸ *a*). Bromoethanol (Chen *et al.*, 2017 ▸) and glycerol (Pan *et al.*, 2012 ▸) have also been mapped in the vestibular cavity of ELIC. Importantly, this cavity seems to be conserved in eukaryotic cationic receptors, as revealed by analysis of the similar region in 5HT3 (Hu *et al.*, 2018 ▸) and the α4β2 acetylcholine receptor X-ray structures (Fig. 5 ▸ *b*). The consensus emerging from the different functional/structural studies in ELIC and sTeLIC is that the intersubunit site would be a negative modulation site and the intrasubunit site a potentiating site. In the case of GLIC, a comprehensive functional analysis of the effect of these carboxylates is required to link binding to modulation. Furthermore, the crystal structures of the same complexes with the closed form of GLIC would be necessary in order to predict the overall activating or inhibiting properties of these compounds, as recently shown for barbiturates (Fourati *et al.*, 2017 ▸). Unfortunately, the closed form of GLIC is very difficult to crystallize and diffracts at best only to a limited resolution (Sauguet *et al.*, 2013 ▸).

At this point, it is tempting to speculate that the existence of two regulatory pockets that are rather close in space might be an evolutionary remnant of a primordial catalytic activity in the extracellular domain of pLGIC likely involving amino-acid metabolism. More specifically, the direct catalytic decarboxylation of an amino acid might have been possible at some point during evolution of the pLGIC family in the vestibular site, with the product diffusing rapidly to the other (ortho­steric) binding site, resulting in the coupled opening of the pore. Indeed, several agonists of pLGICs are directly derived from the simple decarboxylation of amino acids or derivatives thereof (GABA, 5HT3, histamine *etc.*).

## 5. Conclusion   

In conclusion, GLIC can accommodate monocarboxylate or dicarboxylate derivatives in two distinct sites of the extracellular domain. Some carboxylates are known to be allosteric inhibitors of GLIC, but others still need to be characterized from a functional point of view. At the molecular level, all of the carboxylates bind to the intersubunit site that partially overlaps the pLGIC orthosteric site, but only acetate, propionate and succinate bind in the intrasubunit vestibular site. This latter site is reported to be a potentiation site in other bacterial pLGICs and is conserved in eukaryotic cationic receptors. This vestibular site is likely to be a key pharmacological site and thus an important target for rational drug design against some neurological disorders involving human pLGICs.

## Supplementary Material

PDB reference: GLIC–glutarate, 6hja

PDB reference: GLIC–malonate, 6hjb

PDB reference: GLIC–crotonate, 6hji

PDB reference: GLIC–succinate, 6hjz

PDB reference: GLIC–fumarate, 6hj3

PDB reference: GLIC–propionate, 6hpp

Supplementary figures. DOI: 10.1107/S205979832000772X/ag5034sup1.pdf

## Figures and Tables

**Table 1:** Structures of the carboxylic acids used in this study

Carboxylic acid	Structure
Propionate	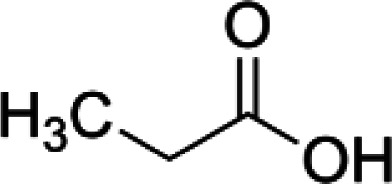
Succinate	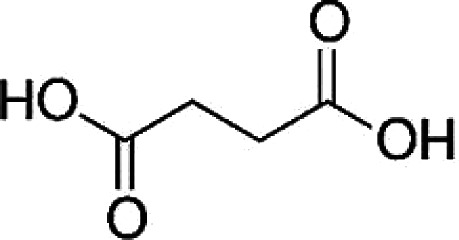
Malonate	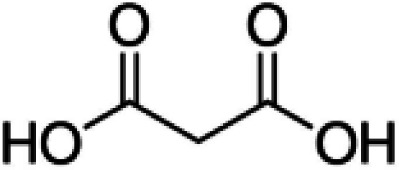
Glutarate	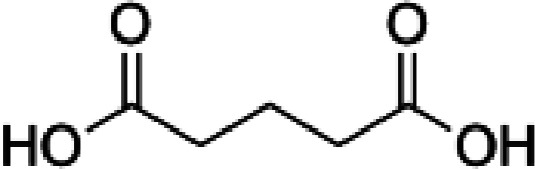
Fumarate	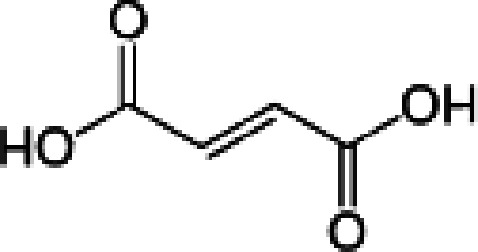
Crotonate	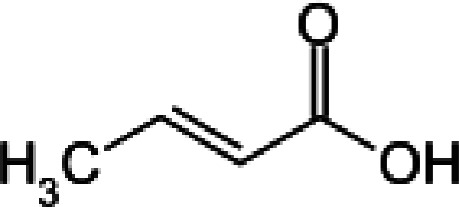

**Table 2:** Data-collection and refinement statistics Values in parentheses are for the highest resolution shell.

Ligand	Propionate	Succinate	Glutarate	Malonate	Fumarate	Crotonate
PDB code	6hpp	6hjz	6hja	6hjb	6hj3	6hji
Wavelength (Å)	0.99	0.91	0.98	0.97	0.97	0.97
Resolution range (Å)	20–3.2	12–2.5	12–2.7	12–3.0	20–2.7	12–2.8
Space group	*C*121	*C*121	*C*121	*C*121	*C*121	*C*121
Unit-cell parameters						
*a* (Å)	180.68	180.71	180.860	180.09	181.510	180.943
*b* (Å)	133.73	132.48	134.412	133.36	132.648	133.242
*c* (Å)	158.89	320.56	160.310	158.98	159.857	159.880
β (°)	101.37	102.22	102.204	101.35	102.083	101.964
Total reflections	221836	881378	355323	312634	511004	632257
Unique reflections	61051	254093	102481	73724	100756	91038
Multiplicity	3.6	3.5	3.5	3.8	5.1	6.9
Completeness (%)	99.8 (99.8)	98.50 (99.02)	99.25 (98.77)	99.72 (99.62)	98.53 (98.23)	99.54 (99.55)
Mean *I*/σ(*I*)	6.6 (0.8)	10.1 (0.9)	13.1 (1.0)	7.8 (1.0)	10.5 (1.0)	9.2 (1.0)
Wilson *B* factor (Å^2^)	90.66	57.14	75.14	80.48	68.14	78.83
*R*_merge_	0.12 (1.76)	0.069 (1.35)	0.048 (1.28)	0.102 (1.31)	0.09 (1.55)	0.136 (1.96)
CC_1/2_	0.99 (0.41)	0.99 (0.61)	0.99 (0.73)	0.99 (0.475)	0.99 (0.62)	0.99 (0.56)
*R*_work_	0.195	0.2337	0.2210	0.2054	0.2125	0.188
*R*_free_	0.201	0.2480	0.2529	0.2217	0.2327	0.204
No. of non-H atoms						
Total	12782	26501	13245	13193	13140	13326
Macromolecule	12625	25258	12670	12654	12634	12729
Ligands	135	1030	458	389	480	470
Solvent	22	213	117	150	26	127
R.m.s.d., bonds (Å)	0.009	0.009	0.01	0.009	0.01	0.009
R.m.s.d., angles (°)	0.97	1.07	1.06	1.02	1.02	1.1
Ramachandran statistics						
Favoured (%)	96.63	97.90	95.73	96.50	96.63	96.96
Allowed (%)	3.37	2.10	4.27	3.50	3.30	3.04
Outliers (%)	0.00	0.00	0.00	0.00	0.06	0.00
Rotamer outliers (%)	7.43	3.82	3.91	4.13	6.07	5.86
Average *B* factors (Å^2^)						
Overall	113.93	75.49	106.73	89.27	89.34	97.55
Macromolecule	113.93	74.76	106.39	88.76	88.92	97.41
Ligands	117.61	94.35	120.07	111.51	101.58	124.09
Solvent	92.41	70.64	90.95	74.20	70.99	71.77
