# Supplementary material for: Structural evidence for the binding of monocarboxylates and dicarboxylates at pharmacologically relevant extracellular sites of a pentameric ligand-gated ion channel
Source: Acta Crystallogr D Struct Biol. 2020 Jun 30;76(Pt 7):668–75. doi: 10.1107/S205979832000772X (PMC7336382; doi:10.1107/S205979832000772X)
Supplement: Supplementary file 1 [file d-76-00668-sup1.pdf]

STRUCTURAL BIOLOGY

Volume 76 (2020)

Supporting information for article:

Structural evidence for the binding of monocarboxylates and dicarboxylates at pharmacologically relevant extracellular sites of a pentameric ligand-gated ion channel

Zaineb Fourati, Ludovic Sauguet and Marc Delarue

A

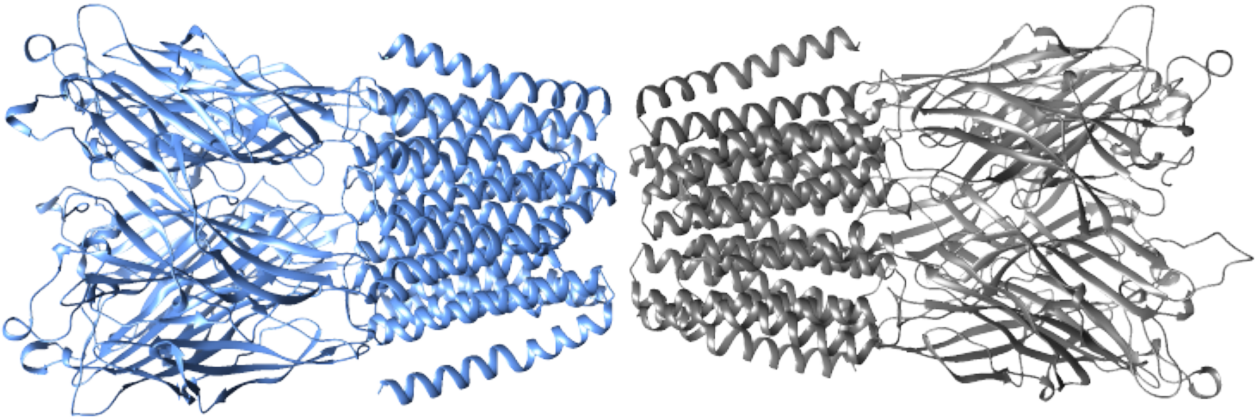

B

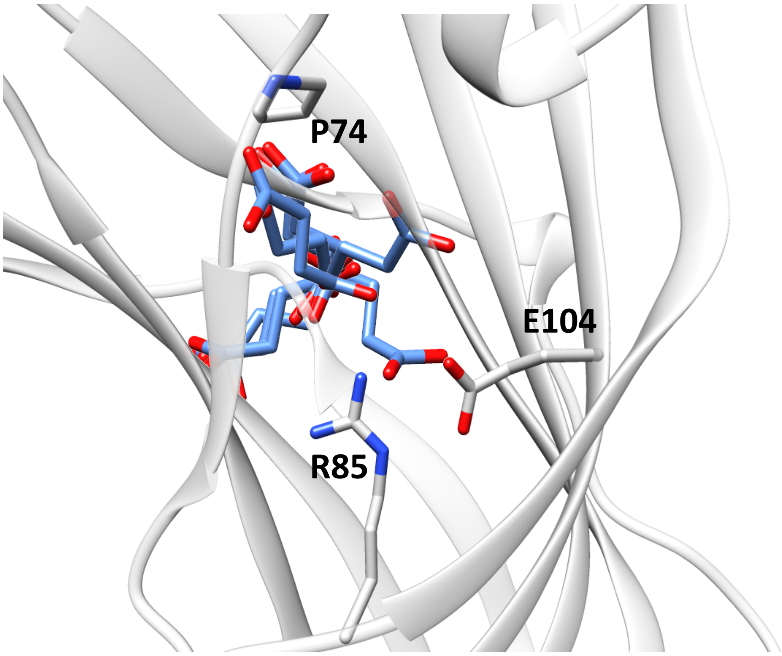

C

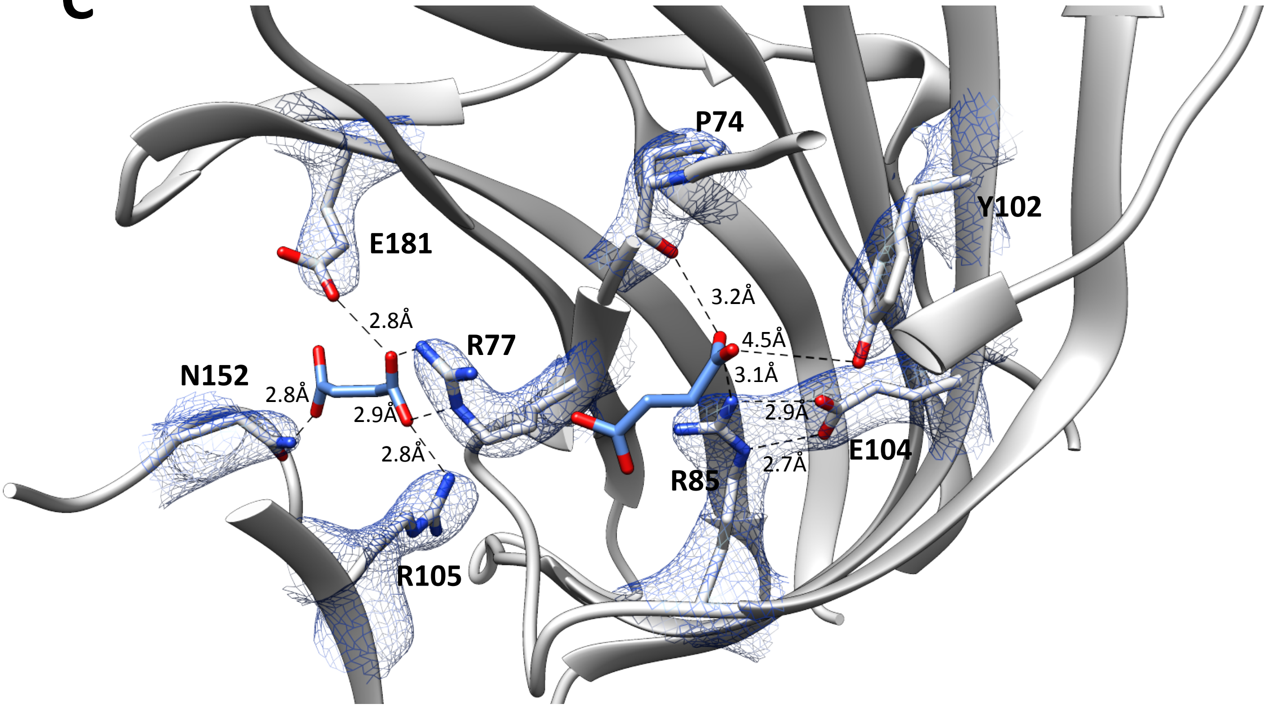

Supplementary figure S1: GLIC7 succinate structure

A- Cartoon representation of the assymetric unit content in succinate bound GLIC structure, where two GLIC receptors are oriented back to back, making contacts through their transmembrane domains.

B- Superimposition of the ten intrasubunit sites found in the two pentamers of the succinate-bound GLIC structure, showing the different binding poses of succinate observed in the intrasubunit site. C- 2Fo-Fc density, contoured at 1σ, of the residues involved in succinate coordination.

Succinate molecules are colored blue.

Intrasubunit site

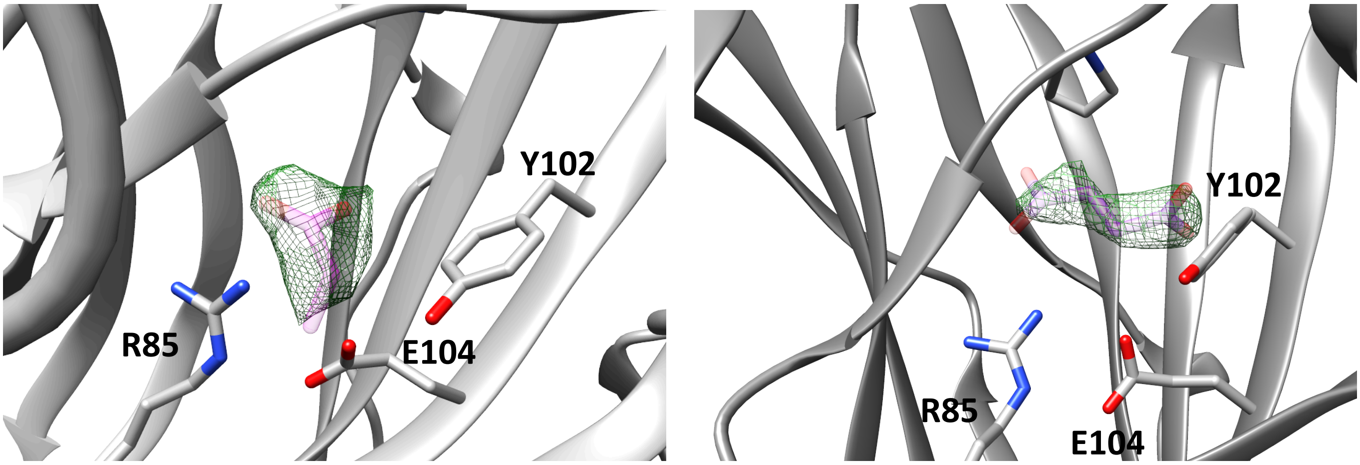

Supplementary figure S2: Representation of the omit Fo-Fc density maps, contoured at 3σ in the *intrasubunit* site of propionate (left) and succinate (right) structures. Propionate (left) and succinate (right) were modelled inside the densities in pink transparent sticks.
